# Cuttlefish Buoyancy in Response to Food Availability and Ocean Acidification

**DOI:** 10.3390/biology9070147

**Published:** 2020-07-01

**Authors:** Eve Otjacques, Tiago Repolho, José Ricardo Paula, Silvia Simão, Miguel Baptista, Rui Rosa

**Affiliations:** MARE—Marine and Environmental Sciences Centre, Laboratório Marítimo da Guia, Faculdade de Ciências, Universidade de Lisboa, Av. Nossa Senhora do Cabo, 939, 2750-374 Cascais, Portugal; tfrepolho@fc.ul.pt (T.R.); jrpaula@fc.ul.pt (J.R.P.); silviazevedo95@gmail.com (S.S.); msbaptista@fc.ul.pt (M.B.); rrosa@fc.ul.pt (R.R.)

**Keywords:** *Sepia officinalis*, cuttlebone, ocean acidification, food availability, calcification, early life stages

## Abstract

Carbon dioxide concentration in the atmosphere is expected to continue rising by 2100, leading to a decrease in ocean pH in a process known as ocean acidification (OA). OA can have a direct impact on calcifying organisms, including on the cuttlebone of the common cuttlefish *Sepia officinalis*. Moreover, nutritional status has also been shown to affect the cuttlebone structure and potentially affect buoyancy. Here, we aimed to understand the combined effects of OA (980 μatm CO_2_) and food availability (fed vs. non-fed) on the buoyancy of cuttlefish newborns and respective cuttlebone weight/area ratio (as a proxy for calcification). Our results indicate that while OA elicited negative effects on hatching success, it did not negatively affect the cuttlebone weight/area ratio of the hatchlings—OA led to an increase in cuttlebone weight/area ratio of fed newborns (but not in unfed individuals). The proportion of “floating” (linked to buoyancy control loss) newborns was greatest under starvation, regardless of the CO_2_ treatment, and was associated with a drop in cuttlebone weight/area ratio. Besides showing that cuttlefish buoyancy is unequivocally affected by starvation, here, we also highlight the importance of nutritional condition to assess calcifying organisms’ responses to ocean acidification.

## 1. Introduction

Human-induced environmental changes pose multiple threats to oceanic ecosystems [1,2]. Oceans are in gas equilibrium with the atmosphere, absorbing around 30% of carbon dioxide (CO_2_) emitted [1]. However, since the industrial revolution, this equilibrium has been threatened by an increase in atmospheric CO_2_ from 280 ppm, during the preindustrial period, to levels exceeding 400 ppm nowadays and the concentration of atmospheric CO_2_ is expected to rise to 730–1020 ppm by the year 2100 [1,3]. This CO_2_, when combined with seawater, forms carbonic acid (H_2_CO_3_), which dissociates into bicarbonate (HCO_3_^−^) and hydrogen ions (H^+^) [2,4], resulting in a consequent decrease in pH—a process known as ocean acidification. Alongside that, the increase in CO_2_ also leads to an increase in bicarbonate, which will later reduce the carbonate ions (CO_3_^2−^) availability in the water [4]. Due to OA-induced carbonate availability reduction, marine calcifying organisms and thus, carbonate-dependent species, are mainly expected to be negatively affected by OA [5,6,7]. The lower CO_3_^2−^ availability directly impacts the calcification of such organisms, as it affects the calcium carbonate (CaCO_3_) deposition and dissolution, used for the establishment of their structures [8]. For example, many tropical corals, planktonic organisms (e.g., pteropods and coccolithophorids), bivalves and echinoderms revealed a reduction in their calcification rates (or an increase in dissolution rates) under OA [7,8,9,10,11,12,13,14,15,16,17].

Besides the impact on carbonate-dependent species, OA is also described to disrupt survival, growth, and physiology and to induce variable behavioral responses in various organisms [18,19,20,21]. In cephalopods, for instance, it has been demonstrated that predatory strategies in squid were altered under high CO_2_, presenting notably an increase in the latency to attack and an alteration of body pattern choice [22]. Moreover, the defensive behaviors and activity of the tropical squid *Idiosepius pygmaeus* seemed to be affected by high CO_2_, where the squids were more active and presented a stronger escape response rather than a defensive arm posture. This could be potentially deleterious regarding predators as it would increase their visibility [23]. Along with their behavioral disruption, it was also shown that exposure to higher levels of CO_2_ led to perturbations in pygmy squid reproduction and embryonic development—e.g., fewer eggs were laid from breeding pairs, with smaller hatchling size [24]. The eggs were as well laid with a reduced space between them, which could further affect the oxygen transfers with the environment [24]. Elevated CO_2_ can also disrupt the development of the squid *Doryteuthis pealeii*, causing an increase in the hatching time, shorter mantle length and the aragonite statoliths used for balance and movements detection were significantly smaller [25,26]. Early life stages of the coastal squid (*Loligo vulgaris*) showed lower embryo survival rate and shorter embryonic periods under OA and warming, as well as an increase in embryo growth rates, premature hatchlings and abnormalities probability [27].

Amongst cephalopods, the cuttlefish is also prone to be affected by OA [28,29]. Both future levels of temperatures and OA were shown to lower the survival rates, shorten the embryonic periods and increase the premature hatching rates of the common cuttlefish *Sepia officinalis* [29]. Moreover, it also affected the perivitelline fluid, highlighting harder conditions inside *S. officinalis* eggs in the future [29]. During embryonic development, cuttlefish form an internal shell, the cuttlebone [30]. The cuttlebone is formed with a stack of calcified chitin lamellae, kept apart by pillars, and thus, forming chambers between them, conferring a skeletal structure to the cuttlefish [30,31]. The cuttlefish is then able to modify the density of its cuttlebone, transferring liquid in and out of the different chambers, through their ventral walls, involving a change in the gas volume present which will subsequently affect the density of the cuttlebone and enable the cuttlefish to regulate its buoyancy [30,31,32,33]. Exposure to elevated *p*CO_2_ was shown to increase by up to 55% the mineralization of CaCO_3_ in *S. officinalis* cuttlebone [34,35,36,37]. Moreover, the structure of the cuttlebone was also modified, with a lamellar spacing reduction of 50%, and an increase in the pillars’ thickness [35]. Although this has been described for higher *p*CO_2_ levels than those expected by the IPCC for 2100, other studies showed similar effects of over-calcification at levels of *p*CO_2_ in accordance with predictions [36,37]. Since changes in cuttlebone density have already been shown to play an important role in the buoyancy of the cuttlefish, the structural changes in cuttlebone due to over-calcification should affect cuttlefish buoyancy [30,35,36,37]. 

Alongside that, cuttlebone structure can also be affected by the organism’s nutritional condition [38]. Cuttlefish under malnutrition have a lower growth rate linked to a reduction in the space between the lamellae [38]. Yet, according to Boletzky, cuttlefish buoyancy capabilities do not appear to be affected by malnutrition, but only with starvation [38,39]. Denton and Gilpin-Brown also found that cuttlefish can display striking differences in buoyancy control capabilities linked to cuttlebone density [30]. Cuttlefish may be: (i) “floaters”—i.e., more buoyant, with lower densities than the seawater, or (ii) “sinkers”—able to rest at the bottom without making any effort [30]. According to these authors, such dichotomy is explained by cuttlebone density differences [30], but the underlying causes are not known. While the effects of food availability on the buoyancy of cuttlefish are relatively unknown, it is worth noting the existence of some related studies in other marine organisms, such as fishes [40,41]. Winter time leads to mass loss in clupeid fishes, where the organic matter is partly replaced by water [42], which is then suggested to affect the fish buoyancy [40]. For instance, the anchovy *Engraulis encrasicolus* displays a differential swimming behavior according to the season—while the anchovy shows a demersal strategy in winter time, it becomes pelagic in spring time. This shift was suggested to be related to a loss in body energy density linked to differential food availability between seasons [41]. These findings linking buoyancy and food availability give some leads towards potential similar effects in other marine organisms, such as cuttlefish.

Here, we aimed to understand, for the first time, the combined effect of food availability (fed vs. non-fed treatments) and ocean acidification (control levels of *p*CO_2_ ~ 400 μatm and future levels of *p*CO_2_ ~ 980 μatm) on cuttlefish (*Sepia officinalis*) newborns’ buoyancy capabilities (and the associated calcification process). We hypothesize that non-fed cuttlefish have disrupted buoyancy control due to under-calcification processes (also translated in a greater proportion of “floaters”) and that changes of buoyancy control might occur due to over-calcification under OA.

## 2. Materials and Methods

### 2.1. Ethical Statement

All the procedures were approved by the FCUL Animal Welfare Committee (ORBEA FCUL) and the Portuguese General-Directorate for Food and Veterinarian Contacts (DGAV) of the Portuguese Government, according to National (Decreto-Lei 113/2013) and the EU legislation (Directive 2010/63/EU) on the protection of animals used for scientific purposes (within the framework of MAR2020—MAR-01.04.02-FEAMP-0007).

### 2.2. Collection and CO_2_ Acclimation Conditions

Recently spawned egg masses of the common cuttlefish *S. officinalis* were collected, in October 2019, in the Sado estuary located on the west coast of Portugal (38°29′18.42” N; 8°53′15.12” O). After collection, the eggs were transported to the aquatic facilities of Laboratório Marítimo da Guia (Cascais, Portugal). The eggs were separated by hand from the different clutches and individually (and randomly) placed in individual vials (a total of 180 eggs were used). After the acclimation time (i.e., 6 days), the eggs were exposed to two different *p*CO_2_ treatments: (1) *p*CO_2_ at present day conditions (control, *n* = 90; *p*CO_2_ ~ 400 μatm, pH = 8.1) or (2) high CO_2_ scenario as expected by the IPCC’s RCP8.5 projections (High CO_2_, *n* = 90; *p*CO_2_ ~ 980 μatm, pH = 7.7) [1], and remained in the same treatment after hatching and until sampling.

A total of 10 transparent tanks of 10 L were used, each of them containing 18 cuttlefish placed in drilled 0.2 L vials; for environmental enrichment, a layer of sand was placed at the bottom of the vial, but not enough for the cuttlefish to bury itself and thus, allowing for a daily monitoring of the cuttlefish status. A lower level of sand was considered in order to keep a good water quality and avoid animal distress and suffering. According to Sykes et al. [43,44], a non-enriched environment is to be considered for hatchlings as it does not affect their growth compared to an enriched environment and shows the advantage to causing less distress due to cleaning processes [43,44]. Distress in organisms was verified daily according to Fiorito et al. [45], for which no such signs have been observed. Each treatment was kept with a semi-open aquatic system. Natural seawater was pumped directly from the sea into a 5 m^3^ seawater storage reservoir and subsequently, filtered to 0.35 µm and UV-irradiated (Vecton 150, TMC Iberia, S. João do Tojal, Portugal), before being supplied to the life support systems. Room illumination, using fluorescent white T8 lights (*n* = 4) at a distance of 1.5 m from the top of the tanks, was kept constant under a photoperiod of 10/14 (light/dark cycle), according to the photoperiod found on the west coast of Portugal in October. To ensure a good seawater quality in the system, the experimental tanks were equipped with bioballs (matured with nitrifying bacteria) for biological filtration. The pH levels were monitored and automatically adjusted every 2 s using a Profilux 3.1 N (GHL, Kaiserslautern, Germany). pH was upregulated by aeration with CO_2_-filtered atmospheric air (soda-line, Sigma-Aldrich, Algés, Portugal), and downregulated with direct injection of CO_2_ gas (Air Liquide, Lisbon, Portugal) in mixing tanks. The temperature was set to 18 °C and regulated with a water bath connected to chillers (Hailea, Guangdong, China). On a daily basis, temperature and pH were monitored manually using a WTW Multi 3510 IDS with a pH-electrode SenTix^®^ 940 (WTW, Weilheim, Germany) and salinity was verified, using a V2 refractometer (TMC Iberia, S. João do Tojal, Portugal). The total alkalinity was calculated once a week, by measuring spectrophotometrically the absorbance of the water at 595 nm (using a Asys UVM 340 microplate reader, Biochrom Ltd., Cambridge, UK). Values of bicarbonate and *p*CO_2_ were calculated with CO2SYS software. The seawater parameters of the experimental setups, as well as a diagram describing the experimental design are summarized in the Supplementary Materials Table S1 and Figure S1, respectively. 

### 2.3. Sampling Endpoints

Cuttlefish were monitored on a daily basis. Once a cuttlefish would hatch, the egg was removed to keep proper water quality. The hatchlings were fed with one gammarid *Gammarus locusta* at a sub-adult or adult stage (from 1000 to 1500 µm and more than 1500 µm, respectively) per day, from a stock production already present at the facility, according to 4 different sampling/feeding treatments: 

Developmental effects:

(1) Newly hatched cuttlefish—the respective cuttlebone was sampled up to 2 days after hatching (DAH) (Control *n* = 22; High CO_2_ *n* = 14); 

Feeding effects:

(2) Non-fed cuttlefish—the respective cuttlebone was sampled once the animal started floating (as previously explained, the cuttlefish was considered as “floater” if it was staying at the surface of the water without moving or unable to reach and remain at the bottom of the vial) (Control *n* = 19; High CO_2_ *n* = 18); 

(3) Fed non-floating cuttlefish—the respective cuttlebone was sampled at a determined DAH paired with the DAH which cuttlefish from the previous treatment started floating (e.g., if a cuttlefish from feeding treatment (2) started floating at DAH 17, we would sample a cuttlefish from this group at DAH 17) (Control *n* = 20; High CO_2_ *n* = 15);

(4) Fed cuttlefish—the respective cuttlebone was sampled after 25 to 30 days (this period of time was longer than for the previous treatments) (Control *n* = 19; High CO_2_ *n* = 15). 

Regardless of feeding treatment (3 or 4), if fed cuttlefish were considered as floaters, they were sacrificed at that time.

### 2.4. Sampling and Analysis of the Cuttlebone

According to the different feeding treatment and as soon as a cuttlefish would be considered as a floater, the cuttlefish was sacrificed (to obtain the respective cuttlebone). To do so, we anesthetized the cuttlefish by placing the specimens first in a solution of 1% MgCl_2_ for 5 min. Then, we increased the dosage to 5% MgCl_2_ to cause overdose for at least 10 min and until complete cessation of activity from the cuttlefish. Confirmation of death by ensuring permanent cessation of circulation was made, in compliance with Directive 2010/63/EU. The cuttlefish was then dissected to collect the cuttlebone. We subsequently took a picture of the wet cuttlebone using a Leica Sapo (Leica Famalicão, Portugal), equipped with a Leica MC190 HD camera (using the LAS V4.12 software), before placing it in an Eppendorf to dry at 60 °C for 12 h. The cuttlebone was then stored until weighing. To make sure that all moisture was removed, cuttlebones were dried a second time for 1 h at 60 °C. The dry cuttlebones were then photographed, as previously explained, and their weight was measured, using a Shimadzu AUW220D scale. Contrarily to Gutowska et al. [35], who studied juvenile cuttlefishes with initial mantle lengths of 27.86 ± 2.13 mm, here, it was not possible to accurately measure cuttlebone thickness due to the small size (and fragility) of newborns’ cuttlebones and thus, having an accurate density measurement. Acknowledging this limitation, our estimates entailed image analysis of dried cuttlebones with the software ImageJ to obtain the area of each cuttlebone and acquire a cuttlebone weight/area ratio.

### 2.5. Statistical Analysis

We performed data exploration, using the protocol suggested by Zuur et al. [46]. Since the time until floating are time-to-event data, we performed a survival analysis to compare the number of days from hatching until floating according to CO_2_ and feeding treatment. We used a proportional hazards Cox mixed effects model fit by maximum likelihood with CO_2_ and feeding treatment as categorical fixed effects. Tank was set as a random factor, as well as the days spent in CO_2_ treatment before hatching; since some individuals have been longer exposed, we added this information in the model as a potential difference explanation. The hatching success was also analyzed with a survival analysis according to the number of days until hatching. We used the CO_2_ treatment as a categorical fixed effect for the proportional hazards Cox mixed effects model fit by maximum likelihood and the tank was set as a random factor. We used the function “coxme” in the package “Coxme” and function “Anova” in the package “car”. We verified compliance with the assumption of proportional hazards using the global test statistic in the function “coxph” from the R package “survival” and graphically using a smoothed spline plot of the Schoenfeld residuals relative to time (Figures S2 and S3). Survival curves were plotted as Kaplan–Meier plots using the function “ggsurvplot” from the R package “survminer”. 

The cuttlebone weight/area ratio was analyzed using generalized linear mixed-models (GLMM) with a Gaussian distribution. To analyze the weight/area ratio at hatching regarding the CO_2_ treatment, we used CO_2_ treatment (factor with two levels—Control and High CO_2_) as the categorical fixed factor, the number of days until hatching as a covariate and tank as a random factor (four individuals were removed from the dataset to perform the analysis as they were unusable for the cuttlebone weight/area ratio, due to the poor cuttlebone state after the sampling). To analyze the cuttlebone weight/area ratio post-hatching (average 18.5 days), we used CO_2_ treatment and feeding treatment (factor with two levels—Fed and Non-fed) as categorical fixed factors and the number of days after hatching as a covariate. Finally, to analyze the cuttlebone weight/area ratio after hatching of fed cuttlefish regarding the floating, we used CO_2_ treatment and floating (factor with two levels—Floater and No-floater) as categorical fixed factors and the number of days after hatching as a covariate. The days until hatching or days after hatching were set as covariate in these models, as individuals were differently exposed to CO_2_ over time and could potentially explain the differences in the model. The full models, with all possible interactions, were tested according to the Akaike Information Criterion (AIC) [47]. Model assumptions such as normality, independence and absence of residual patterns were verified graphically according to Zuur and Ieno [48]. We used the function “lmer” from the package “lme4” and the function “Anova” from the package “Car”. All statistical analyses and graphs were performed in R, version 3.6.2 (R Core Team 2019).

## 3. Results 

**Figure 1:**
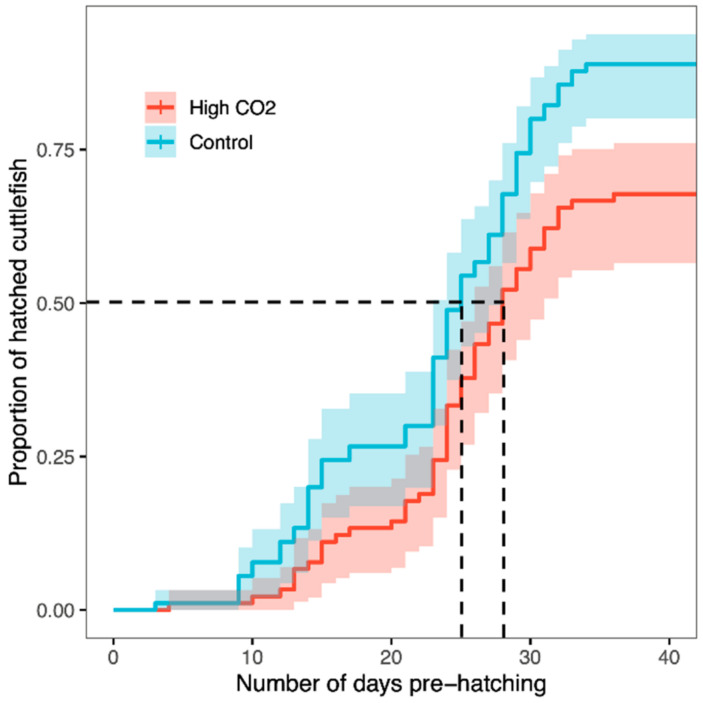
Effects of CO_2_ treatment on cuttlefish hatching success, according to the number of days of exposure. The presence of new hatchlings was verified every 24 h. Kaplan–Meier survival trajectories illustrate the different survival trajectories according to the CO_2_ treatment. Lines represent the rate of hatched cuttlefish at each day of exposure. Shaded areas are 95% confidence intervals. Dashed lines represent the hatching time, defined as the point when 50% of the embryos hatched.

The cuttlefish hatching success over time was significantly different according to the CO_2_ treatment, namely, the control hatching success was 88.9%, with 22.44 ± 7.38 (mean ± std. dev.) number of days until hatching and high CO_2_ hatching success was 68.9%, with 24.16 ± 6.83 number of days until hatching (*n_total_* = 180, *n_hatched_control_* = 80, *n_hatched_HighCO2_* = 62, *n_non-hatched_control_* = 10, *n_non-hatched_HighCO2_* = 28; χ^2^ = 4.221; d.f. = 1, *p* = 0.040; Tables S2 and S3, Figure 1). In fact, the hatching time (here, defined as the point when 50% of the embryos hatched) was 25 days and 28 days, under control and high CO_2_, respectively (see dashed vertical lines in Figure 1). 

**Figure 2:**
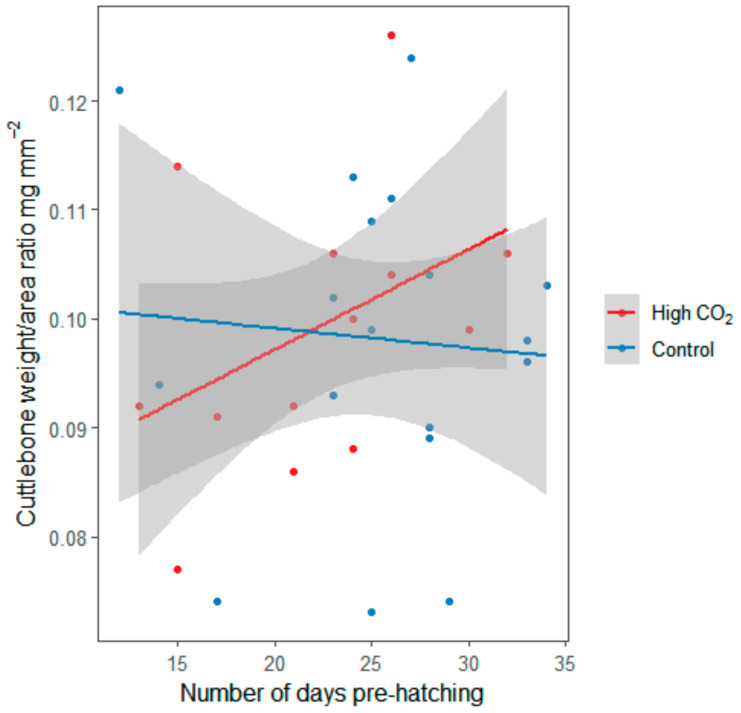
Effect of CO_2_ treatment on hatchlings’ cuttlebone weight/area ratio, according to the number of days of exposure, before hatching. Lines represent the linear regression of the cuttlebone weight/area ratio, according to the CO_2_ treatments. Points represent the raw data. Shaded areas are 95% confidence intervals.

However, the cuttlebone weight/area ratio at hatching was not altered by pre-hatching CO_2_ exposure (control = 0.098 ± 0.015 mg mm^−2^; high CO_2_ = 0.099 ± 0.13 mg mm^−2^; *n* = 36, χ^2^ = 0.369; d.f. = 1; *p* = 0.544; Tables S4 and S5, Figure 2). 

**Figure 3:**
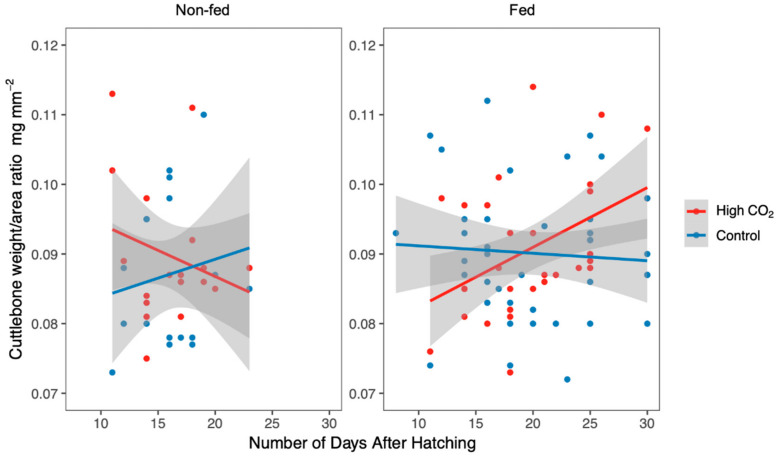
Effect of CO_2_ treatment and feeding treatment (regrouped into two groups—Fed and Non-fed) on newborns’ cuttlebone weight/area ratio, according to the number of days of exposure, after hatching. Lines represent the linear regression of the cuttlebone weight/area ratio according to the CO_2_ treatments. Points represent the raw data. Shaded areas are 95% confidence intervals.

Later on, newborns cuttlebone weight/area ratio showed a significant three-way interaction between CO_2_, feeding and days in treatment after hatching, where the average weight/area ratio for control–fed individuals was 0.090 ± 0.010 mg mm^−2^, for control–non-fed individuals was 0.086 ± 0.011 mg mm^−2^, for high CO_2_–fed individuals was 0.091 ± 0.010 mg mm^−2^ and for high CO_2_–non-fed individuals was 0.090 ± 0.010 mg mm^−2^ (*n* = 106, χ^2^ = 4.034; d.f. = 1; *p* = 0.045; Tables S6 and S7, Figure 3). For instance, under OA, cuttlebone weight/area ratio showed opposing trends between non-fed (negative) and fed (positive) cuttlefish with exposure time (Figure 3).

**Figure 4:**
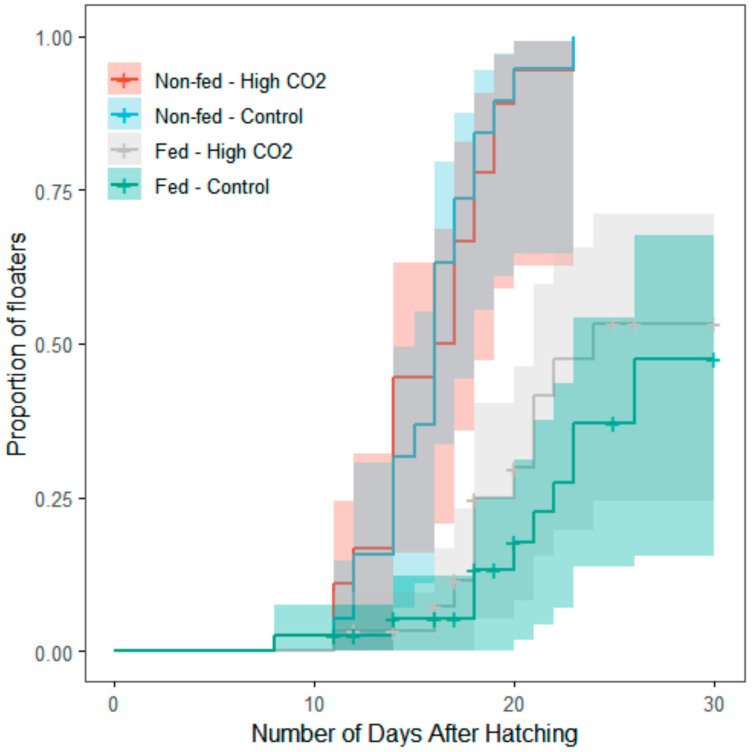
Effect of feeding treatment (regrouped into two groups—Fed and Non-fed) on the buoyancy of newborns cuttlefish, according to the number of days of exposure, after hatching. The presence of floaters was verified every 24 h. Kaplan–Meier survival trajectories illustrate the different survival trajectories according to the CO_2_ treatment and feeding treatment. Lines represent the rate of floaters cuttlefish at each day of exposure. Shaded areas are 95% confidence intervals.

There was no significant interaction between CO_2_ exposure and feeding treatment regarding floating rate (χ^2^ = 0.970; df = 1; *p* = 0.325). Yet, the proportion of floaters was significantly different between feeding treatments (fed = 0.304 ± 0.464; non-fed = 1.000 ± 0.000; χ^2^ = 47.085; d.f. = 1; *p* < 0.001) but not within CO_2_ treatments (control = 0.500 ± 0.504; high CO_2_ = 0.604 ± 0.494; *n* = 106, χ^2^ = 0.430; d.f. = 1; *p* = 0.512; Tables S8 and S9, Figure 4). 

**Figure 5:**
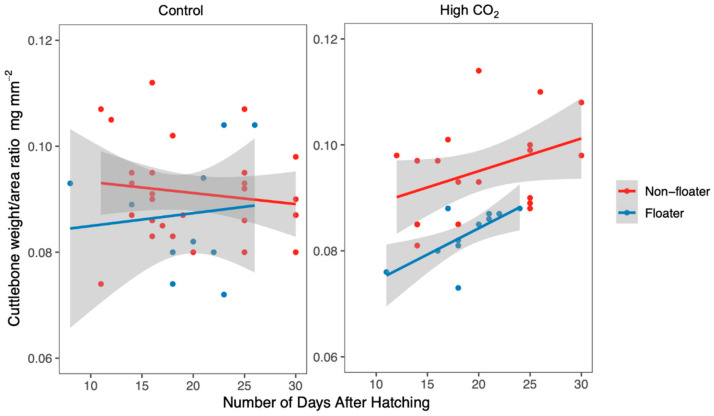
Effect of CO_2_ treatment and buoyancy on fed newborns’ cuttlebone weight/area ratio, according to the number of days of exposure, after hatching. Lines represent the linear regression of the cuttlebone weight/area ratio according to the state of buoyancy of the cuttlefish. Points represent the raw data. Shaded areas are 95% confidence intervals.

Regarding fed cuttlefish, there was a significant difference in the cuttlebone weight/area ratio according to the interaction between CO_2_ treatment and exposure time after hatching (average weight/area ratio in control = 0.090 ± 0.010 mg mm^−2^; high CO_2_ = 0.091 ± 0.010 mg mm^−2^; χ^2^ = 3.928; d.f. = 1; *p* = 0.0475) and floating rate (floaters = 0.085 ± 0.009, non-floaters = 0.093 ± 0.009; *n* = 69, χ^2^ = 11.460; d.f. = 1; *p* < 0.001; Tables S10 and S11, Figure 5). 

## 4. Discussion

OA is known to differently affect calcification processes [20]. Contrarily to previous studies [36,49], here, we observed that cuttlefish hatching success was significantly lower under high CO_2_ according to the exposure time. More specifically, hatching success was lower when the cuttlefish spent a longer time of their embryonic development under high CO_2_, with a delay of three days regarding the hatching time (here, defined as the time when 50% of cuttlefish hatched) when compared to control treatment. Since we show that longer-exposed cuttlefish embryos have lower hatching success under OA, this suggests that cuttlefish at later embryonic developmental stages are more tolerant to high CO_2_ than earlier stages. This could indicate that CO_2_ might be affecting earlier processes, such as gastrulation [50], as only the cuttlefish that spent earlier developmental processes in high CO_2_ were affected. Furthermore, these findings can be related to differences in early ontogenetic acid-base regulation capabilities linked to deficient functionality of ion regulatory epithelia during early embryogenesis [51]. 

While pre-hatching exposure to high CO_2_ had a significant effect on the hatching success, this did not seem to significantly affect the cuttlebone weight/area ratio of the hatchlings. This result is consistent with a previous study showing that lower pH did not affect the weight of the cuttlebone at hatching [36]. Yet, Dorey et al. [36] showed a calcium accumulation increase of 17% in cuttlefish hatchlings [36]. Therefore, we cannot rule out the possibility of over-calcification (and enhanced calcium accumulation) in hatchlings under high CO_2_ since the cuttlebone weight/area ratio is only a rough proxy for calcification. As suggested by Dorey et al. [36], high CO_2_ could alter the size, the internal structure and organization of the cuttlebone, leading to an increase in calcium accumulation without affecting the weight, or the weight/area ratio, in our case [36]. In the present study, a few weeks after hatching, we already found differences in cuttlebone weight/area ratio of newborns. Non-fed cuttlefish had lower cuttlebone weight/area ratio under high CO_2_, whereas fed cuttlefish presented an increased cuttlebone weight/area ratio over time under high CO_2_, which is consistent with the findings of Gutowska et al. [35], where cuttlefish (also fed in that study) presented an increase in cuttlebone mass related to an increase in mineralized CaCO_3_ [35]. On the other hand, our results show that OA-driven over-calcification processes are highly dependent on food availability. Ramajo et al. [52] and Brown et al. [53] also found that OA-disruption of calcification processes is dependent on food availability on other calcifying species, such as the mussel *Mytilus edulis* or the brittlestar *Amphiura filiformis* [52,53,54,55,56]. *A. filiformis* exposed to high CO_2_ also presented an increase in calcification, yet it came along with a decrease in arm muscle mass, suggesting a trade-off between the arm function and the integrity of the skeleton [54]. *M. edulis* was also able to mitigate the negative effects of high CO_2_ on the calcification process under a favorable feeding regime [52,53,55,56]. In our study, only fed cuttlefish presented a higher cuttlebone weight/area ratio under high CO_2_, and we advocate that such OA-associated over-calcification can offset or overcompensate possible CO_2_ dissolution effects on the cuttlebone under low food availability regimes. This notion supports the hypothesis that food availability may modulate the effects of climate change [34,35,36,37,52,53,57] and that usual ad libitum feeding might mask potential variation in the effects of OA [57].

It has been suggested that cuttlefish raised under high CO_2_ conditions (with induced over-calcification) may display some impairments in swimming abilities and buoyancy control [34,35,36]. Although the present findings did not corroborate such an idea, it is worth noting that this study only used newborn cuttlefish up to 30 days old. Further long-term acidification-related studies should be carried out as the cuttlebone functional properties could be further affected later on in the ontogenetic development. 

Concomitantly, here, we observed that the food availability had a significant impact on the buoyancy, as all starved cuttlefish floated. As with previous studies suggesting that buoyancy was not affected by malnutrition but only starvation [38,39], we observed here that starvation had a significant negative impact on the buoyancy control of cuttlefish. Non-fed and “fed floaters” cuttlefish showed lower cuttlebone weight/area ratios compared to non-floaters (“sinkers”), which may be due to modifications in the cuttlebone structure. Regarding the “fed floaters”, it is worth mentioning that such an unexpected occurrence in the feeding treatment was associated with the fact that some newborns revealed a lack of predatory action towards the gammarids and did not feed. As previously said, malnutrition can affect the structure of the cuttlebone, with a decrease in its size due to reduced lamellae spacing, but not buoyancy per se [38]. The space reduction between septum has also been suggested in studies with extinct cephalopods (Carboniferous ammonoids), probably due to changes in the environment, including low oxygen and low food availability [58,59]. It is worth noting that, according to Sherrard [60], besides the Nautilus group, cuttlefish of the *Sepia* genus might also be considered a modern-day analog to ammonoids in terms of buoyancy control capabilities [60]. As already mentioned, malnutrition results in shorter chambers in *Sepia* [38], which is a feature that may allow the cuttlebone to be more resistant to implosion [60,61]. This could also explain seasonal changes in depth use of *S. officinalis* when feeding is reduced in deeper waters during the winter [32,60]. However, we argue that, whereas cuttlefish can deal with malnutrition for a certain period of time [60], if malnutrition is prolonged with starvation, this would lead to a decrease in cuttlefish’s ability to control buoyancy and, subsequently, their ability for predation. 

## 5. Conclusions

Here, we showed that although OA had negative effects on the hatching success, there was no significant difference in cuttlebone weight/area ratio at the hatchling stage. However, signs of increased cuttlebone weight/area ratio were only found in fed cuttlefish exposed to high CO_2_. This CO_2_ effect related to food availability is added to previous studies of marine invertebrate’s responses to OA such as the barnacle *Amphibalanus improvisus* or the mussel *Mytilus edulis* that both showed abilities to withstand CO_2_ stress when food availability was high [56,62]. Moreover, it was shown that OA can also impact an organism due to deleterious effects on its feed nutritional quality, showing that OA can induce severe impacts on marine food webs in different ways [63]. Thus, the present findings, in addition to these previous studies, highlight the importance of accounting for food availability to accurately determine calcifying organisms’ responses to ocean acidification.

## Data Availability

The datasets and analyzed during this study are available in the Figshare repository, 10.6084/m9.figshare.11942136.

## Supplementary Materials

The following are available online at https://www.mdpi.com/2079-7737/9/7/147/s1, Table S1: Seawater physicochemical parameters in all experimental setups. Figure S1: Diagram describing the experimental design. Figure S2: Smoothed spline plots of Schoenfeld residuals of the hatching success Cox mixed effects model relative to time. Figure S3: Smoothed spline plots of Schoenfeld residuals of the floating Cox mixed effects model relative to time. Table S2: Summary output for Cox mixed effects model of hatching success among CO_2_ treatments. Table S3: Analysis of deviance table (Type II tests) for the Cox mixed effects model on the hatching success among CO_2_ treatments. Table S4: Summary output for mixed effects model of cuttlebone weight/area ratio of hatchlings among CO_2_ treatments and days in treatment until hatching. Table S5: Analysis of deviance table (Type II tests) for the mixed effects model of cuttlebone weight/area ratio of hatchlings among CO_2_ treatments and days in treatment until hatching. Table S6: Summary output for mixed effects model of cuttlebone weight/area ratio of newborns among CO_2_ treatment, feeding treatment and days in treatment after hatching. Table S7: Analysis of deviance table (Type II tests) for the mixed effects model of cuttlebone weight/area ratio of newborns among CO_2_ treatments and days in treatment until hatching. Table S8: Summary output for Cox mixed effects model on floating among CO_2_ and feeding treatments. Table S9: Analysis of deviance table (Type II tests) for the Cox mixed effects model on floating among CO_2_ and feeding treatments. Table S10: Summary output for mixed effects model of cuttlebone weight/area ratio of newborns among CO_2_ treatment, floating and days in treatment after hatching. Table S11: Analysis of deviance table (Type II tests) for the mixed effects model of cuttlebone weight/area ratio of newborns among CO_2_ treatments, floating and days in treatment after hatching.
