# Supplementary material for: Cuttlefish Buoyancy in Response to Food Availability and Ocean Acidification
Source: Biology (Basel). 2020 Jul 1;9(7):147. doi: 10.3390/biology9070147 (PMC7407613; doi:10.3390/biology9070147)
Supplement: Supplementary file 1 [file biology-09-00147-s001.pdf]

Supplemental materials

Cuttlefish buoyancy in response to food availability and ocean acidification

Eve Otjacques *, Tiago Repolho, José Ricardo Paula, Silvia Simão, Miguel Baptista and Rui Rosa

MARE—Marine and Environmental Sciences Centre, Laboratório Marítimo da Guia, Faculdade de Ciências, Universidade de Lisboa, Av. Nossa Senhora do Cabo, 939, 2750-374 Cascais, Portugal ; tfrepolho@fc.ul.pt (T.R.); jrpaula@fc.ul.pt (J.R.P.); silviazevedo95@gmail.com (S.S.); msbaptista@fc.ul.pt (M.B.); rrosa@fc.ul.pt (R.R.)

* Correspondence: ebotjacques@fc.ul.pt

Table S1. Seawater physicochemical parameters in all experimental setups.

	Control	High CO_2
Measured		
Temperature (ºC)	18.3 ± 0.2	18.4 ± 0.2
pH	8.059 ± 0.005	7.743 ± 0.033
TA (µmol/kgSW)	2620.4 ± 13.5	2640.1 ± 29.8
Salinity (ppm)	36.0 ± 0.1	36.0 ± 0.1
Calculated		
TC (µmol/kgSW)	2331.4 ± 10.8	2508.6 ± 27.5
pCO2 (µatm)	426.7 ± 6.0	994.8 ± 79.6
Ω Arg	3.3 ± 0.1	1.8 ± 0.1

Salinity, pH and temperature were measured daily and averaged over the whole experimental period. The combination of total alkalinity (TA) and pHT (pH total scale) was used to calculate carbonate system parameters [pCO_2 (carbon dioxide partial pressure), TC (total inorganic carbon) and Ω Arg (aragonite saturation state)]. Values are represented as mean ± standard deviation

Figure S1. Experimental design and timeline according to Sampling Endpoints.

Recently spawned egg masses were collected in October 2019 in Sado estuary (Portugal).
The egg were separated by hand and individually placed in vials.

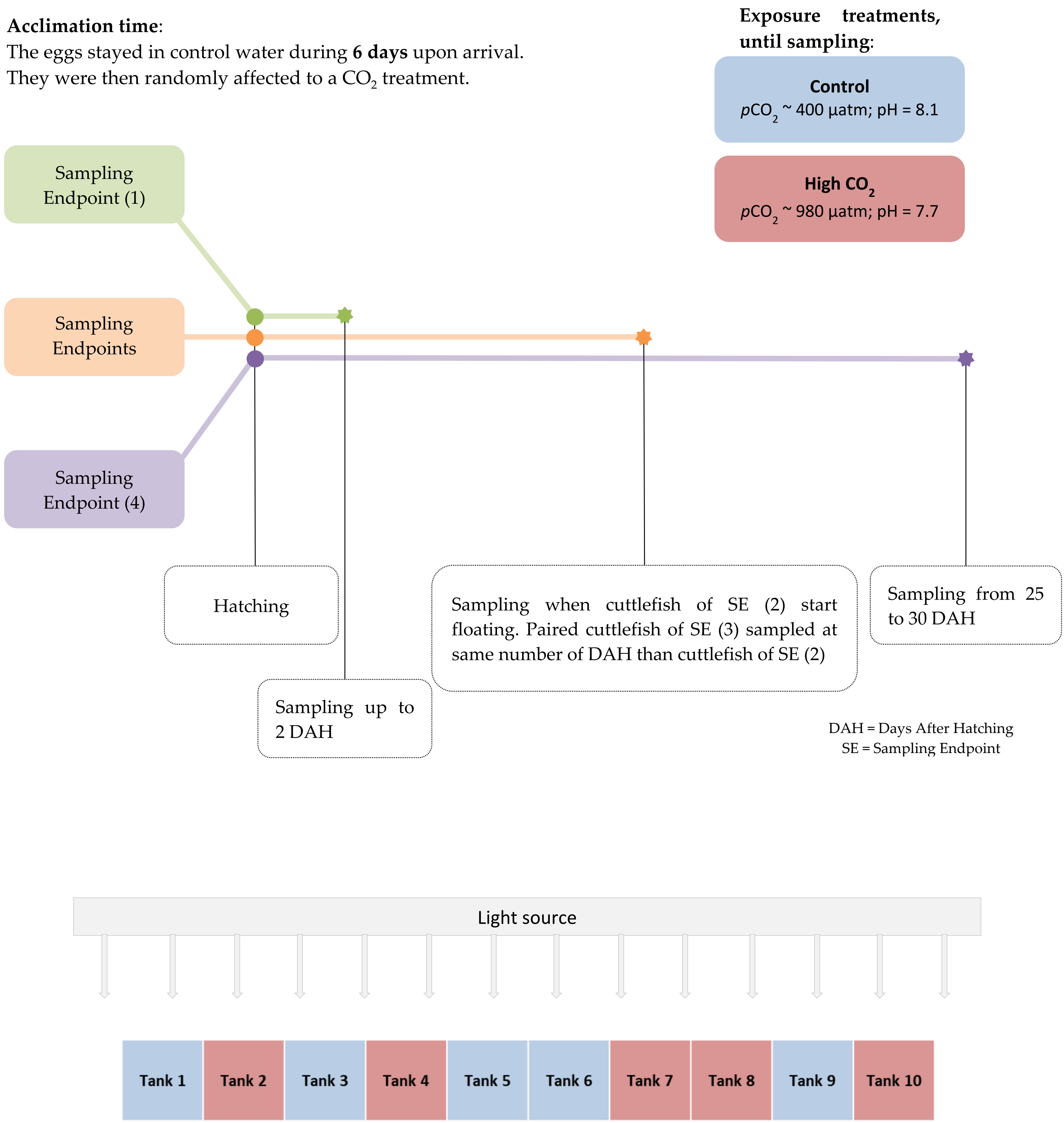

Figure S2. Smoothed spline plots of Schoenfeld residuals of the hatching success Cox mixed effects model relative to time.

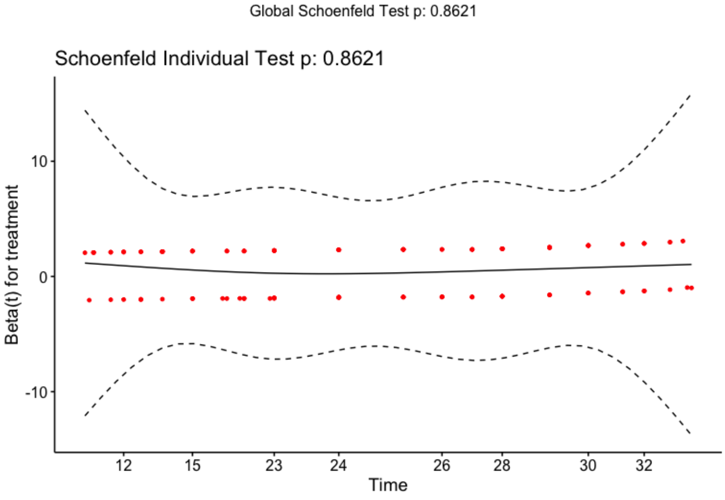

Figure S3. Smoothed spline plots of Schoenfeld residuals of the floating Cox mixed effects model relative to time.

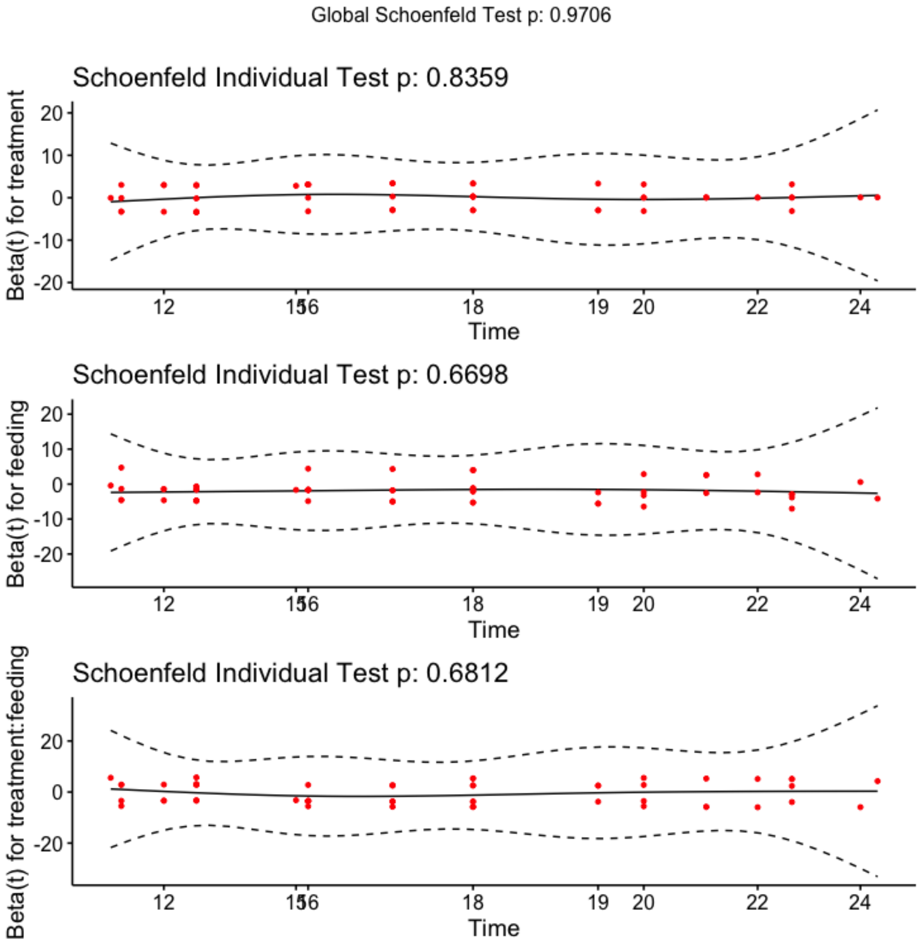

Table S2. Summary output for Cox mixed effects model of hatching success among CO_2 treatments.

	**coef**	**exp(coef)**	**se(coef)**	**z**	***p***
CO_2 treatment $_{control}$	0.542	1.719	0.264	2.050	**0.040**

Table S3. Analysis of deviance table (Type II tests) for the Cox mixed effect model on the hatching success among CO_2 treatments. Values of p<0.05 are shown in bold.

	**df**	**χ^2**	***p***
CO_2 treatment	1	4.220	**0.040**

Table S4. Summary output for mixed effects model of cuttlebone weight/area ratio of hatchlings among CO_2 treatments and days in treatment until hacthing.

	**Estimates**	**Std. error**	**t-value**	***p***
(Intercept)	0.079	0.015	5.011	**<0.001**
CO_2 treatment $_{control}$	0.024	0.021	1.117	0.264
Days until hatching	0.001	0.001	1.345	0.179
CO_2 treatment $_{control}$ × days until hatching	-0.001	0.001	-1.242	0.214

Table S5. Analysis of deviance table (Type II tests) for the mixed effects model of cuttlebone weight/area ratio of hatchlings among CO_2 treatments and days in treatment until hatching. Values of p<0.05 are shown in bold.

	**df**	**χ^2**	***p***
CO_2 treatment	1	0.135	0.713
Days until hatching	1	0.369	0.544
CO_2 treatment × days until hatching	1	1.543	0.214

Table S6. Summary output for mixed effects model of cuttlebone weight/area ratio of newborns among CO_2 treatment, feeding treatment and days in treatment after hatching.

	Estimates	Std. error	t-value	*p*
(Intercept)	0.102	0.012	8.436	**<0.001**
Feeding	-0.028	0.014	-1.971	**0.049**
CO_2 treatment $_{control}$	-0.026	0.018	-1.473	0.141
days_until_sampling	-0.001	0.001	-1.014	0.311
Feeding × CO_2 treatment $_{control}$	0.045	0.020	2.222	**0.026**
Feeding × days until sampling	0.002	0.001	1.948	0.051
CO_2 treatment $_{control}$ × days until sampling	0.001	0.001	1.290	0.197
Feeding × CO_2 treatment $_{control}$ × days until sampling	-0.002	0.001	-2.009	**0.045**

Table S7. Analysis of deviance table (Type II tests) for the mixed effects model of cuttlebone weight/area ratio of newborns among CO_2 treatments and days in treatment until hatching. Values of $p<0.05$ are shown in bold.

	df	χ^2	*p*
Feeding	1	0.622	0.430
CO_2 treatment $_{control}$	1	0.830	0.362
days_until_sampling	1	0.858	0.354
Feeding × CO_2 treatment $_{control}$	1	1.445	0.229
Feeding × days until sampling	1	0.579	0.447
CO_2 treatment $_{control}$ × days until sampling	1	2.164	0.141
Feeding × CO_2 treatment $_{control}$ × days until sampling	1	4.034	**0.045**

Table S8. Summary output for Cox mixed effects model on floating among CO_2 and feeding treatments.

	**coef**	**exp(coef)**	**se(coef)**	**z**	***p***
CO_2 treatment $_{control}$	0.024	1.025	0.346	0.070	0.940
Feeding	-1.996	0.136	0.426	-4.680	**<0.001**
Feeding × CO_2 treatment $_{control}$	-0.562	0.570	0.570	-0.980	0.320

Table S9. Analysis of deviance table (Type II tests) for the Cox mixed effect model on floating among CO_2 and feeding treatments. Values of $p<0.05$ are shown in bold.

	**df**	**χ^2**	***p***
CO_2 treatment	1	0.430	0.512
Feeding	1	47.085	**<0.001**
Feeding × CO_2 treatment	1	0.970	0.325

Table S10. Summary output for mixed effects model of cuttlebone weight/area ratio of newborns among CO_2 treatment, floating and days in treatment after hatching.

	**Estimates**	**Std. error**	**t-value**	***p***
(Intercept)	0.084	0.008	10.949	**<0.001**
Floating	-0.022	0.017	-1.283	0.200
CO_2 treatment $_{control}$	0.012	0.010	1.252	0.211
Days until sampling	0.001	0.000	1.587	0.112
Floating × CO_2 treatment $_{control}$	0.006	0.021	0.278	0.781
Floating × days until sampling	0.001	0.001	0.616	0.538
CO_2 treatment $_{control}$ × days until sampling	-0.001	0.000	-1.798	0.072
Floating × CO_2 treatment $_{control}$ × days until sampling	0.000	0.001	0.053	0.958

Table S11. Analysis of deviance table (Type II tests) for the mixed effects model of cuttlebone weight/area ratio of newborns among CO_2 treatments, floating and days in treatment after hatching. Values of p<0.05 are shown in bold.

	df	χ^2	*p*
Floating	1	11.460	**0.001**
CO_2 treatment $_{control}$	1	0.459	0.498
Days until sampling	1	0.648	0.421
Floating × CO_2 treatment $_{control}$	1	2.351	0.125
Floating × days until sampling	1	1.240	0.266
CO_2 treatment $_{control}$ × days until sampling	1	3.928	**0.047**
Floating × CO_2 treatment $_{control}$ × days until sampling	1	0.003	0.958
